# Objectively Assessed Physical Activity and Subsequent Health Service Use of UK Adults Aged 70 and Over: A Four to Five Year Follow Up Study

**DOI:** 10.1371/journal.pone.0097676

**Published:** 2014-05-27

**Authors:** Bethany Simmonds, Kenneth Fox, Mark Davis, Po-Wen Ku, Selena Gray, Melvyn Hillsdon, Debbie Sharp, Afroditi Stathi, Janice Thompson, Joanna Coulson, Tanya Trayers

**Affiliations:** 1 Centre for Exercise, Nutrition and Health Sciences, School for Policy Studies, University of Bristol, Bristol, United Kingdom; 2 Graduate Institute of Sports and Health, National Changhua University of Education, Changhua City, Changua, Taiwan; 3 Department of Health and Applied Social Sciences, University of the West of England, Bristol, United Kingdom; 4 Sport and Health Sciences, Exeter University, Exeter, United Kingdom; 5 The School of Social and Community Medicine, The University of Bristol, Bristol, United Kingdom; 6 Department for Health, The University of Bath, Bath, United Kingdom; 7 School of Sport & Exercise Sciences, University of Birmingham, Birmingham, United Kingdom; University of California, San Francisco, United States of America

## Abstract

**Objectives:**

To examine the associations between volume and intensity of older peoples' physical activity, with their subsequent health service usage over the following four to five years.

**Study Design:**

A prospective cohort design using baseline participant characteristics, objectively assessed physical activity and lower limb function provided by Project OPAL (Older People and Active Living). OPAL-PLUS provided data on numbers of primary care consultations, prescriptions, unplanned hospital admissions, and secondary care referrals, extracted from medical records for up to five years following the baseline OPAL data collection.

**Participants and Data Collection:**

OPAL participants were a diverse sample of 240 older adults with a mean age of 78 years. They were recruited from 12 General Practitioner surgeries from low, middle, and high areas of deprivation in a city in the West of England. Primary care consultations, secondary care referrals, unplanned hospital admissions, number of prescriptions and new disease diagnoses were assessed for 213 (104 females) of the original 240 OPAL participants who had either consented to participate in OPAL-PLUS or already died during the follow-up period.

**Results:**

In regression modelling, adjusted for socio-economic variables, existing disease, weight status, minutes of moderate-to-vigorous physical activity (MVPA) per day predicted subsequent numbers of prescriptions. Steps taken per day and MVPA also predicted unplanned hospital admissions, although the strength of the effect was reduced when further adjustment was made for lower limb function.

**Conclusions:**

Community-based programs are needed which are successful in engaging older adults in their late 70s and 80s in more walking, MVPA and activity that helps them avoid loss of physical function. There is a potential for cost savings to health services through reduced reliance on prescriptions and fewer unplanned hospital admissions.

## Introduction

The value of physical activity for the maintenance of physical and cognitive function and the prevention of coronary heart disease, stroke, diabetes and some cancers is now well established [1]–[2]. The presence of these chronic illnesses and non-communicative diseases in older age is associated with higher levels of medication and health care usage, and in particular, unplanned hospital admissions [3]–[4]. Given that the older sector of the population is expanding and living longer, it follows that physical activity promotion, as a means of preventing further disease has great potential to reduce these burgeoning health care costs.

Surprisingly, research on the health service usage (HSU) of older adults is sparse, particularly for those already in their late 70s and 80 s where usage is likely to be greatest, and for a nationalised healthcare system such as that found in the United Kingdom. In the United States and Australia, research examining health service utilisation is usually based on insurance claim data which is cost-focused [5]–[6]. Other studies have relied on self-reported usage and visit information collected through questionnaires [7]–[8]. This method has been shown to be unreliable [9]–[11]. The few studies that have examined the medical records of older patients to establish their health usage have focused on number of hospital admissions or number of accident and emergency admissions only [12]–[13].

Sari [14] provided a recent review of 19 survey and intervention studies addressing the links between physical activity and healthcare utilization in people aged 65 and over. Results provided supportive evidence to show that higher physical activity is associated with lower healthcare usage. However, the author drew attention to limitations in design such as biased samples, non-equivalent control groups, and reliance on self-report measures suggesting that findings are not generalisable to a wider ageing population. He concluded that longitudinal studies with representative samples using robust measurement and statistical methods were needed to take the field forward. Our further searches have revealed no further studies addressing the prospective link between physical activity levels and subsequent health service usage of older adults.

A measurement challenge exists with the assessment of physical activity in older adults. Most of their activity is in the form of daily walking for shopping and visiting friends or family and these events are less memorable than structured physical activity [15], such as attending exercise classes or playing sports. Self-report may be subject to bias from factors such as socially desirable responding [16], or lack of precision due to poor recall [17]. Objective measures such as accelerometry also have some limitations such as incapacity to identify type and context of activity, or accurately assess some activities such as cycling and swimming [18]. However, accelerometry produces high resolution data which can be used to record temporal patterns of activity, and estimate volumes of activity at different intensities. This is useful as there are indications from the existing evidence base that activity that is of at least moderate intensity provides most of the health benefit [1]–[2]. Objective measures of physical function are also important as they are able to provide more robust estimates of capacities important for everyday living such as balance, coordination, strength and mobility, than self-report measures [19].

Physical activity and aspects of physical function are closely related. Low activity can contribute to mobility deterioration over time and higher activity can improve or maintain mobility [20]. Conversely, mobility limitations, which might arise from conditions such as osteoarthritis or heart conditions can also have a detrimental effect on physical activity and may have effects on some health outcomes such as hip fracture that are independent of activity [21]. It is therefore important to provide robust assessments of both physical activity and function when investigating health usage outcomes. Consequently, this study with UK adults aged 70 and over, provided objective measures of both physical activity and lower limb function at baseline and estimates of health service usage and new disease diagnosis, derived from primary care records, over the following four to five years. The specific purpose of the research was to examine the independent prospective associations between a) total volume of activity and b) volume of activity at higher intensity, and subsequent health service usage, while adjusting for potential confounding variables.

## Methods

### Study design

This paper combines data from Project OPAL (Older People and Active Living) and OPAL-PLUS (How have you been?) a follow-up study. OPAL was an observational cohort study, conducted in 2006/8, of 240 adults aged 70 and over. The objectives of OPAL were to provide comprehensive objective documentation of physical activity and lower limb function and their determinants and consequences in a diverse cohort of UK city-dwelling older people aged 70 and over. OPAL-PLUS involved a further comprehensive assessment three years later. As a sub study of OPAL-PLUS, we also extracted health service usage data from primary care records for up to five years following the baseline OPAL data collection.

### Ethics statement

Ethics approval for OPAL and OPAL-PLUS was obtained from Bristol Southmead NHS REC (Ethics reference 06/Q2002/127). Informed written consent was given by all OPAL participants who also agreed to access to their GP records.

### Participants

OPAL participants were initially recruited from 12 General Practitioner (GP) surgeries in the West of England, stratified by low, medium or high Index of Multiple Deprivation (IMD) of the patient catchment area and low or high access to local amenities. The OPAL sample was selected at random from each patient list following screening by a GP for: 1) recent bereavement, 2) terminal illness, 3) debilitating mental illness, 4) inability to complete a questionnaire, and 5) any other illness preventing participation. The final OPAL sample consisted of 115 females (48%) with a mean age of 79 (±6) years, and 125 males with a mean age of 77 (±6) years. The age and gender of the sample differed minimally from the patient lists from which they were selected. The percentages of either overweight or obese were similar to those from national samples: 66% of men and 57% of women [22]. Additionally, the IMD of the OPAL sample was diverse with 22.1%, 27.9%, 31.3%, and 18.7% falling in the lowest to highest national quartiles in England respectively [23]. For this paper, we report HSU data collected from OPAL participants GP records over the following four to five years.

### OPAL baseline variables

OPAL baseline data were collected through two home visits and these have been described in detail elsewhere [24]–[25]. For this paper, the following OPAL baseline demographic data were collected as they have established associations with both health and physical activity [22]: age group (70.0–74.9, 75.0–79.9, 80.0–84.9 and ≥85.0 years), gender, highest level of education attained (primary, secondary or tertiary) and IMD of area of residence. Numbers of existing illness and conditions already being treated were also collected through a standardised structured interview. Body Mass Index (BMI) was calculated from measured height and weight and participants allocated to weight status groups (obese - >30.0 kg·m2; overweight −25.0–29.9 kg·m2; and normal/underweight−<25.0 kg·m2).

#### Physical activity

Seven-day accelerometry was used to objectively measure physical activity, for more detail, see Davis *et al*. [24]. Data inclusion required at least 10 hours of monitoring on at least five days. The following daily summary variables: registered wear time (hours•d-1), total physical activity (counts per registered minute [CPM]), and minutes of moderate-to-vigorous physical activity (MVPA) (>1951 CPM) and total steps per day (STEPS) were calculated using Kinesoft (v3.3.62; Kinesoft, Saskatchewan, Canada; www.Kinesoft.org).

#### Lower limb function

Participants' lower limb function was assessed using the Short Physical Performance Battery (SPPB) which includes home-based tests of leg strength, walking speed, and balance [19]. Scores for these three measures were summed to produce a lower limb function score (range 0–12).

### HSU variables

An instrument was required that was capable of capturing and summarising several aspects of health service usage over a four to five year period. Our literature search revealed several studies where HSU had been recorded using single measures such as number of consultations, number of hospitalisations, number of medications, number of specialist appointments, and number of new diagnoses of illness [12]–[13]as indicators. No standardised and validated comprehensive recording instrument was identified, so we developed, piloted, and modified a comprehensive proforma that accommodated the four recording systems encountered among the 12 practices. This proforma was designed to record the following variables:

#### Consultations

Number of face-to-face consultations, telephone calls, treatment room appointments and home visits involving at least one primary health care professional.

#### Unplanned hospital admissions

Number of urgent or unplanned hospital admissions, including admissions to an accident and emergency (A and E) unit, ascertained through attached hospital discharge letters.

#### Referrals to secondary care

Number of referrals General Practitioners (GPs) made on behalf of participants to secondary health care services for further treatment.

#### Prescriptions

Number of prescriptions written by GPs for each drug or medical product.

#### Diagnoses of Quality and Outcomes (QOF) conditions

Number of new diagnoses of the chronic illnesses/conditions required for the QOF in primary care. These include asthma, atrial fibrillation, cancer, cardiovascular disease/hypertension, chronic kidney disease, COPD, coronary heart disease, dementia, depression, diabetes, epilepsy, heart failure, hypothyroidism, other mental illness, obesity and palliative care. We also added rheumatoid arthritis because it will be included in 2014's QOF and has relevance to physical activity and function.

To minimise inconsistency, HSU data were collected from each practice by the same post-doctoral researcher in October 2012. Mean time lapse since OPAL baseline assessment was 50 (±14) months. The GP surgeries used four different data management systems. Four practices used a standard GP management system (EMIS LV), four used an updated windows-based version (EMIS Web), three used Synergy, and one practice used Vision, both web-based systems. EMIS Web and Synergy were the most comprehensive and able to provide more detailed data on prescriptions, consultations, referrals and chronic conditions. To accommodate these differences, we adjusted for GP practice recording system in regression analyses.

### Statistical methods

The sample size of the original OPAL study was powered for cross-sectional group comparisons. For this prospective study, therefore, we conducted a post-hoc power analysis using Version 11.0 of Power Analysis and Sample Size Software (NCSS 2011, Kaysville, UT) for negative binomial regression models. Our sample size of 213 participants achieved, at a p<0.05 significance level, 84–88% power to detect an incidence rate ratio of 1.40 (the average response rate ratios based on our fully adjusted models) with the mean exposure time of 4.29 years. These power estimations included adjustment for R-squared and over-dispersion parameters. The listwise deletion method was used for handling missing data which occurred for MVPA (2.3% of sample), STEPS (5.6%) and education (3.8%). Where variables were combined in regression, missing data for STEPS models was 8.9% and for MVPA was 5.6%.

Lower limb function was split into three criterion-based groups (low ≤6, medium 7–9, high 10–12) based on scores which have previously correlated with tests of mobility disability [19]. In the absence of validated criteria for activity volume assessed by accelerometry, physical activity variables were grouped into tertiles. IMD was split into quartile groups for compatibility with previous publications.

Annual numbers for each HSU variable from baseline to follow-up data collection for those still registered at their practice, or to their recorded date of death (n = 33), or their move to another practice (n = 15) were calculated. T-tests or ANOVAs were then used to assess significance of differences among each of the socio-demographic, BMI, IMD, activity and function groups. Chi Square was used to test for group differences in percentage of each group diagnosed with a new disease during follow-up.

We chose STEPS rather than CPM as the more powerful indicator of total volume of activity, and MVPA to represent volume of activity at higher intensity, for inclusion as two independent variables in regression analysis. Each of these was regressed separately on each of the four HSU variables: a) number of consultations, b) number of unplanned hospital admissions, c) number of referrals to secondary care, and d) number of prescriptions.

Two adjusted models were calculated for each of these regressions. Model 1 was adjusted for the natural logarithm of elapsed time to follow-up, and also age, gender, educational attainment, IMD, weight status and total number of diseases/conditions reported at baseline, which are all known to be associated with physical activity [22]. Additionally, using the fixed-effects method we adjusted for the four GP data management systems that we encountered as the means of the HSU measures varied. Model 2 added adjustment for our objective measure of lower limb function which we regarded as a confounding variable. Because of the close association between physical activity and function and the potential of both to contribute to health status, this provided an opportunity to see the strength of relationship of activity, independently of an important aspect of physical function.

Following the recommendation of Sari's study and recent and relevant review of literature linking physical activity and healthcare utilization [14 26], we adopted negative binomial regression as the statistical method of choice for these regression models. This procedure can accommodate count data, even when they are positively skewed and over-dispersed, as is usually the case with HSU variables. The regression coefficients (B) are not directly interpretable in this method, because of the nonlinearity of the negative binomial distribution; therefore, the incident rate ratios (IRRs) (eB) are presented. For example, if the IRR for number of prescriptions predicted by MVPA (low vs. high) is 1.95, the average number of prescriptions among participants with the lowest level of MVPA is 1.95 times higher than those with highest level of MVPA. In order to compensate for multiple comparisons, we considered Bonferroni adjustment for the four models (alpha = 0.05/4 = 0.0125) so that IRRs were considered statistically significant when p-value was smaller than 0.0125. All analyses were performed with IBM SPSS 20.0.

## Results

**Figure 1:**
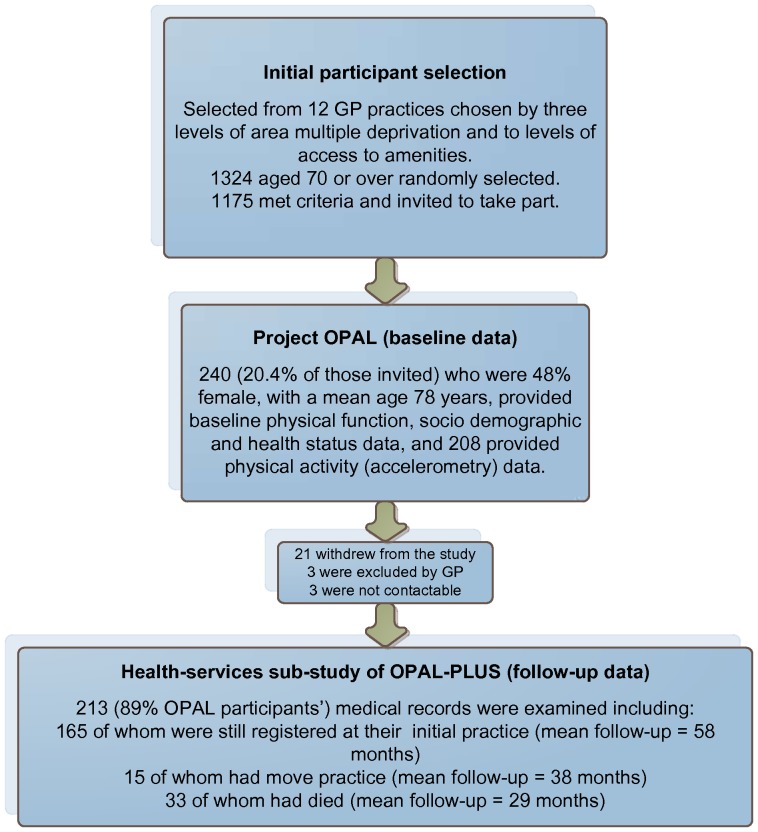
Participant selection and involvement.

Figure 1 shows patient selection and flow. We were able to extract HSU data for 213 of the original 240 OPAL participants. Of the 27 lost participants, three were withdrawn by their GPs as being unsuitable for the OPAL-PLUS follow-up, 21 withdrew by choice, and three were not contactable. Of the remaining 213 cases, all had provided complete socio-demographic (with the exception of 8 missing cases for level of educational attainment), BMI, and existing disease/condition and lower limb function data at baseline. Accelerometry data was available for 208 of them (7 of these did not provide step data due to technical error). There were no statistically significant differences in any socio-demographic variables between the full baseline sample (n = 240) and those whose records were examined (n = 213).

During follow-up 33 participants passed away, with 17 deaths occurring in the first two years, giving an average follow-up of HSU data of 29 (±19) months. Of the 180 participants who were alive during follow-up, 15 had moved GP practice and therefore HSU data were only available until the date that their move was recorded. Length of follow-up for those still alive and registered at the same practice (n = 165) was 58 (±5) months, and 38 (±18) months for those who had moved practice (n = 15).

### Participant characteristics and health service usage

Table 1 displays the mean follow-up time, the percentage of participants who had at least one new disease diagnosed during follow-up, and mean annual numbers of each HSU variable for age, gender, educational attainment, IMD and weight status groups. There were significant differences (p = 0.017) between age groups for follow-up time, which can be explained by higher death rates in the older groups. No statistically significant group differences in any of the HSU means emerged for any of these variables. A total of 132 (62%) participants had at least one new disease diagnosed during follow-up, but similarly, there were no group differences.

**Table 1:** Baseline participant characteristics, percentage with a new disease diagnoses and HSU means during follow up.

Variable groupings	Average follow up time in months (SD)	% (N) with new disease diagnosis	Mean (SD) number of consultations/y	Mean (SD) number of prescriptions/yr	Mean (SD) number of unplanned hospital admissions /y	Mean (SD) number of secondary care referrals/y
**Age (n = 213)**						
70–74.9 (n = 78)	54 (11)	62 (48)	14.4 (10.7)	9.9 (13.9)	1.0 (1.4)	1.1 (1.1)
75–79.9 (n = 57)	52 (14)	63 (36)	15.1 (10.7)	9.4 (13.9)	0.8 (0.9)	1.3 (1.2)
80–84.9 (n = 53)	50 (16)	66 (35)	16.0 (9.0)	6.8 (4.8)	0.9 (1.1)	1.6 (1.3)
85–89.9 (n = 25)	44 (20)	52 (13)	17.3 (11.0)	10.0 (8.8)	1.2 (1.2)	1.3 (1.4)
	p = 0.017	p = 0.690	p = 0.620	p = 0.468	p = 0.463	p = 0.248
**Gender (n = 213)**						
Female (n = 104)	53 (14)	64 (66)	15.4 (9.9)	8.4 (8.8)	0.9 (1.2)	1.4 (1.2)
Male (n = 109)	50 (14)	61 (66)	15.2 (10.8)	9.6 (14.0)	0.9 (1.2)	1.2 (1.2)
	p = 0.405	p = 0.662	p = 0.895	p = 0.462	p = 832	p = 0.350
**Educational attainment (n = 205)**						
Primary (n = 41)	52 (14)	71 (29)	16.0 (11.4)	10.3 (13.7)	0.8 (1.2)	1.1 (1.1)
Secondary (n = 66)	49 (16)	68 (45)	15.2 (9.9)	7.8 (8.2)	0.9 (1.2)	1.3 (1.2)
Tertiary (n = 98)	53 (12)	55 (54)	14.7 (9.7)	9.0 (12.5)	1.0 (1.2)	1.4 (1.3)
	p = 0.286	p = 0.394	p = 0.749	p = 0.462	p = 0.761	p = 0.267
**IMD (n = 213)** 1						
Low (n = 53)	52 (15)	33 (62)	16.8 (11.8)	9.2 (12.2)	0.8 (1.1)	1.2 (1.1)
Low-Med (n = 53)	51 (13)	34 (65)	15.2 (11.1)	10.1 (1.7)	0.9 (1.3)	1.1 (1.0)
Medium (n = 52)	49 (17)	34 (62)	15.3 (9.5)	6.7 (6.5)	0.9 (1.1)	1.5 (1.3)
High (n = 55)	54 (12)	31 (58)	14.1 (8.8)	9.9 (14.3)	1.0 (1.2)	1.3 (1.3)
	p = 0.505	p = 0.912	p = 0.603	p = 0.868	p = 0.868	p = 0.350
**BMI (n = 213)**						
Normal/underweight (n = 73)	52 (12)	60 (44)	14.9 (9.2)	13.6 (1.6)	1.2 (0.2)	1.0 (0.1)
Overweight (n = 80)	53 (14)	59 (47)	15.7 (10.7)	10.2 (1.1)	1.1 (0.1)	1.3 (0.1)
Obese(n = 60)	49 (17)	68 (41)	15.3 (11.2)	11.2 (1.4)	1.3 (0.2)	1.3 (0.2)
	p = 0.407	p = 0.479	p = 0.878	p = 0.505	p = 0.940	p = 0.965

1 IMD, Index of Multiple Deprivation-scores derived from participants' postcodes (high score indicates high level of deprivation).

### Physical activity, lower limb function and health service usage

Table 2 shows the mean for each of the CPM, STEPS, and MVPA, and lower limb function groups. Also displayed for each group are the mean follow-up time, the number of self-reported diseases/conditions at baseline, numbers of new diseases diagnosed during follow-up, and each of the HSU variable means. Significant differences emerged between CPM, STEPS, MVPA and lower limb function groups in numbers of diseases/conditions registered at baseline. Low active groups had significantly more baseline diseases (p<0.001). Numbers of existing diseases clearly may affect subsequent health service usage and was therefore adjusted in the regression models.

**Table 2:** Follow-up time, existing disease at baseline, % (n) with new disease diagnoses, and HSU variables by physical activity and function groups.

Variable and groups	Group mean (SD)	Average follow up time in months (SD)	Mean (SD) number of diseases at baseline	% (N) with new disease diagnosis	Mean (SD) number of consultations/y	Mean (SD) number of prescriptions/y	Mean (SD) number of unplanned hospital admissions/y	Mean (SD) number of secondary care referrals/y
**Counts per minute(CPM)**								
Low (n = 69)	83.3 (30.0)	47 (19)	1.8 (1.3)^a^	65.2 (45)	17.7 (11.2)^b^	12.0 (13.4)^b^	1.3 (1.4)^a^	1.4 (1.4)
Medium (n = 70)	166.8 (20.7)	53 (11)	1.2 (0.9)	60.0 (42)	13.3 (7.9)	7.2 (7.7)	0.8 (1.0)	1.2 (1.0)
High (n = 69)	306.3 (121.0)	54 (11)	1.2 (1.2)	58.0 (40)	14.7 (11.0)	7.7 (12.5)	0.6 (1.0)	1.1 (0.9)
	**p<0.001**	**p = 0.010**	**p = 0.001**	p = 0.666	**p = 0.034**	**p = 0.025**	**p = 0.003**	p = 0.257
**Steps per day (STEPS)**								
Low (n = 64)	2067.7 (784.6)	47 (18)^a^	1.8 (1.2)^a^	68.8 (44)	17.5 (11.4)	12.8 (14.3)^a^	1.4 (1.4)^a^	1.4 (1.4)
Medium (n = 67)	4196.3 (574.2)	54 (13)	1.2 (1.0)	62.7 (42)	13.8 (8.9)	7.5 (7.9)	0.7 (0.9)	1.2 (1.1)
High (n = 70)	7065.8 (1936)	55 (8)	1.1 (1.1)	51.4 (36)	14.4 (10.5)	7.0 (11.6)	0.7 (1.1)	1.1 (0.8)
	**p<0.001**	**p = 0.003**	**p = 0.001**	p = 0.112	p = 0.088	**p = 0.006**	**p = 0.001**	p = 0.332
**Mod-vig mins/day (MVPA)**								
Low (n = 69)	3.3 (2.2)	47 (18)^b^	1.8 (1.2)^a^	69.8 (48)	17.5 (10.7)^d^	12.0 (13.7)^a^	1.4 (1.4)^a^	1.4 (1.3)
Medium (n = 70)	14.4 (3.1)	55 (12)	1.2 (1.0)	58.6 (41)	14.9 (9.4)	7.8 (7.2)	0.8 (1.0)	1.2 (1.1)
High (n = 69)	38.7 (22.5)	52 (10)	1.1 (1.1)	55.1 (38)	13.2 (10.4)	7.1 (12.5)	0.6 (1.0)	1.1 (1.0)
	**p<0.001**	**p = 0.005**	**P<0.001**	p = 0.190	**p = 0.043**	**p = 0.030**	**P<0.001**	p = 0.326
**Lower limb function**								
Low (27)	5.0 (1.3)	43 (20)^a^	1.6 (1.2)	66.7 (18)	15.2 (8.3)	12.4 (13.3)	1.6 (1.3)^d^	1.3 (1.4)
Medium (48)	8.2 (0.9)	48 (18)	1.9 (1.3)^c^	77.1 (37)	18.7 (11.0)^c^	12.5 (14.9)^c^	1.1 (1.4)	1.8 (1.5)^c^
High (138)	11.2 (0.8)	54 (11)	1.2 (1.0)	55.8 (77)	14.1 (10.3)	7.2 (9.6)	0.7 (1.0)	1.1 (1.0)
	**p<0.001**	**P<0.001**	**P<0.001**	**p = 0.028**	**p = 0.032**	**p = 0.007**	**p = 0.001**	**p = 0.006**

a) Low significantly different to medium and high group.

b) Low significantly different to medium group.

c) Medium significantly different to high group.

d) Low significantly different to high group.

There were differences in HSU means between activity groups. Those lowest in CPM (p = 0.003), STEPS (p = 0.001), and MVPA (p<0.001) had more unplanned hospital admissions, and prescriptions than medium or high active groups. Group differences were also seen for lower limb function where secondary referrals (p = 0.006), and prescriptions (p = 0.007) were higher in the medium than high function group. The lowest function group had significantly more unplanned hospital admissions than the high function group (p = 0.001).

Tables 3 and 4 show summaries of the regression results for the prediction of number of consultations, prescriptions, unplanned hospital admissions, and secondary referrals by STEPS and MVPA. Model 1 is adjusted for time lapse to follow-up, socio-demographic characteristics, weight status, existing disease at baseline and GP recording system. Model 2 adds in adjustment for the objective measure of lower limb function. As found in many other studies, physical activity and lower limb function were closely related. For the 213 participants, zero-order correlations between STEPS, and MVPA with lower limb function were r = 0.55 and 0.44 respectively so it was important to isolate the confounding effects of this variable. There were no significant associations between STEPS and MVPA for either primary care consultations (Table 3) or secondary referrals (Table 4). However, a significant effect was seen for MVPA in Model 1 with greater numbers of prescriptions for the low group compared to the high group (IRR [CIs]  = 1.53[1.18–2.00]) and also the medium MVPA group compared to the high group (IRR [CIs]  = 1.41[1.09–1.84]). This effect remained undiminished in Model 2 with the adjustment of lower limb function.

**Table 3:** Negative binomial regression predicting use of consultations and prescriptions based on measures of physical activity.

	IRRs for number of consultations		IRRs for number of prescriptions	
	Model 1	Model 2	Model 1	Model 2
**STEPS**	*p* = 0.652	*p* = 0.591	*p* = 0.169	*p* = 0.491
Low	1.15 (0.83–1.32)	1.08 (0.83–1.39)	1.27 (0.97–1.66)	1.18 (0.89–1.56)
Medium	0.95 (0.78–1.17)	0.96 (0.78–1.17)	1.06 (0.82–1.36)	1.06 (0.82–1.36)
High (ref)	1.00	1.00	1.00	1.00
**MVPA**	*p* = 0.164	*p* = 0.113	***p*** ** = 0.006**	***p*** ** = 0.009**
Low	1.25 (0.99–1.57)	1.29 (1.02–1.64)	1.53 (1.18–2.00)	1.47 (1.12–1.93)
Medium	1.15 (0.92–1.44)	1.16(0.92–1.45)	1.41 (1.09–1.84)	1.45 (1.12–1.87)
High (ref)	1.00	1.00	1.00	1.00

IRR (incidence rate ratio), which is defined as *e* ^B^, where B is the regression coefficients.

**Model 1**: adjusting a baseline covariates, including age, gender, educational attainment, IMD tertile, weight status, and number of self-reported chronic illnesses, and also GP Management System, and time lapse to follow-up;

**Model 2**: additionally adjusting for lower limb function.

**Table 4:** Negative binomial regression predicting unplanned hospital admissions and referrals based on measures of physical activity.

	IRRs for number of unplanned admissions		IRRs for number of referrals to secondary care	
	Model 1	Model 2	Model 1	Model 2
**STEPS**	*p* = 0.069	*p* = 0.394	*p* = 0.909	*p* = 0.834
Low	1.81 (1.09–3.01)	1.36 (0.79–2.34)	0.94 (0.69–1.27)	0.91 (0.66–1.24)
Medium	1.41 (0.85–2.33)	1.37 (0.85–2.20)	0.96 (0.75–1.23)	0.06 (0.75–1.23)
High (ref)	1.00	1.00	1.00	1.00
**MVPA**	***p*** ** = 0.007**	*p* = 0.062	*p* = 0.733	*p* = 0.566
Low	2.13 (1.26–3.60)	1.85 (1.09–3.13)	0.99 (0.75–1.33)	0.99 (0.73–1.31)
Medium	1.28 (0.74–2.21)	1.26 (0.73–2.15)	1.10 (0.84–1.44)	1.12 (0.85–1.47)
High (ref)	1.00	1.00	1.00	1.00

An effect for hospital admissions was seen for MVPA in Model 1 where the low group were at twice the risk of admissions than the high MVPA group (IRR [CIs]  = 2.13[1.26–3.60]). This effect remained although was diminished with the additional adjustment of lower limb function in Model 2(IRR [CIs]  = 1.85[1.09–3.13]).

## Discussion

This study is the first to attempt to provide an estimation of the impact of physical activity, independently of lower limb function, on subsequent HSU for a cohort of UK older adults who were mostly in their late 70s and 80s. Its unique contributions are: a) the objective assessment of physical activity and lower limb function, b) the development of a comprehensive profile of HSU indicators taken from primary records, c) a summary of records across a four to five year time span, d) adjustment in regression models for nine confounding variables including existing disease and objectively assessed lower limb function.

In this diverse cohort living in urban and suburban localities varying from low to high deprivation, there were no relationships between educational attainment, IMD, new disease diagnoses and the HSU variables of number of primary care consultations, number of prescriptions, unplanned hospital admissions or referral to secondary health services. There was no evidence therefore, of greater health service reliance due to lower levels of socio-economic or educational status. Also, there were no significant patterns of HSU differences by age group, gender or weight status. These findings are contrary to several large epidemiological studies that do show differences by gender, age and socio-economic group [27]–[29]. We suggest that this may be a result of these factors becoming less predictive in older age (baseline mean age of 78 years) when patterns of disease and decline may have become more evenly distributed across socio-economic groups.

The number of steps taken per day or the volume of higher intensity activity does not appear to have an independent influence on either primary care consultations or referrals to secondary services. We observed that consultation rates varied considerably from practice to practice and so the relationship may be dominated by the way the practice operates or its accessibility. The previous evidence base demonstrates the impact of physical activity and function on consultations and referrals is equivocal and has relied on self-report measures [14]. The only other study using an objective measure did not find a relationship between cardiovascular fitness assessed using a treadmill and frequency of primary care visits [30]. However, as indicated by one recent well-designed trial, it may be possible for interventions to improve fitness through increases in activity and this may result in reduced consultations in primary care [31].

In contrast, physical activity taken at higher intensity is predictive of numbers of medical prescriptions. This effect shows a relationship with both low and medium MVPA groups receiving approximately 50% more prescriptions than the high MVPA group. This effect remained strong after adjustment for all variables including lower limb function. We could find no other studies against which to compare this effect. MVPA is an indicator of higher metabolic and circulatory load and has been the element of physical activity most strongly related to reduced risk of conditions and diseases common in older age such high blood pressure, type 2 diabetes, coronary heart disease, and stroke [1]–[2], all which are managed long term through medication.

Both MVPA and STEPS were predictive of higher prevalence of unplanned hospital admissions. The more powerful effect was seen once again for MVPA where the low MVPA group was almost twice as likely to be vulnerable than the high MVPA group. However, the significance of the effect was lost when lower limb function was taken into account. This may at least in part be explained by the relationship between physical function and incidence of falls and fractures which are the main cause of emergency admissions in older adults [21 32].

## Implications

A current key public health issue is the rising cost of health and social care of the older population. The identification of a cost-effective means of increasing quality of life, independence, mobility, good health, and social engagement of older adults has now become a priority [33]–[34]. Leading a physically active life has well-established health benefits. This study suggests that it may also have economic benefits by reducing reliance on medications and assisting in the prevention of costly emergency hospital admissions.

The emergence of the importance of MVPA in these relationships is in line with the evidence base that informs physical activity guidelines [1]–[2]. This indicates that the main disease-related benefits are seen with 30 minutes of activity of at least moderate intensity. For this study, greater than 23 minutes per day of MVPA was required to be included in the high MVPA group (mean 39 minutes) which is roughly in line with the 30 minute activity recommendation. However, our measure of MVPA included all minutes of MVPA and not only those minutes accumulated in prolonged bouts of 10 minutes or more which feature in recommendations. Just over 4000 steps per day were sufficient to avoid the lower tertile of STEPS. These values seem achievable for healthy older adults.

Susceptibility to unplanned hospital admissions was greatest among those with poor lower limb function so maintaining scores on the SPPB of 7 or more appears to be an important risk reduction strategy. Indeed, programs such as the LIFE pilot project in USA show that physical activity interventions can reverse or delay progression towards mobility disability [18], [20], [35], especially when they incorporate a muscle strengthening element [36].

The evidence presented here provides further support for the recent guidance from the National Institute for Health and Clinical Excellence recommendations that GPs should be encouraging physical activity in their older patients at a level appropriate for their current levels of activity, function and health [37]. The data also strengthen the case for increasing the availability of and access to community-based programs designed to encourage daily moderate-to-vigorous activity among older adults. Findings also highlight the need for centre-based exercise provision targeting and tailored for older adults in their 70s and 80s, which aim to increase muscle strength, balance, coordination and aerobic fitness and the prevention of falls. These could be modified to provide therapeutic programs for those who already have limited lower limb function, perhaps as a result of a fall or debilitating disease.

## Limitations

There were limitations to this study. The sample size of 213 is small compared to most prospective cohort studies. Small sample sizes increase the risk of missing a true positive effect. We feel that this is unlikely to be the case for the unexpected but consistently low correlations between socio-demographic and HSU variables. Also, when significant results are indicated with small samples, there is a tendency for the effect sizes to be inflated [38]. Our positive findings for activity with HSU variables appeared to be logical and robust but may be susceptible to this inflation. As a result of our criterion referencing, there were small numbers of participants in the low function group and this may have reduced capacity to detect some group differences. Some caution therefore needs to be applied when interpreting results and replication with larger samples, but which also retain objective measures should be pursued. Categorisation of different levels of intensity of physical activity by accelerometers requires the use of cut-points. Our quantification of MVPA is based on a cut point of movement counts that is derived from limited data with older adults and the level of misclassification is not known. The measure should be regarded as an estimate of activity at higher intensity. There were exclusions for: 1) recent bereavement, 2) terminal illness, 3) debilitating mental illness, 4) inability to complete a questionnaire, and 5) any other illness preventing participation. This may have limited the range of activity and function and constrained predictive effects. It is known that older adults who volunteer for physical activity studies tend to be healthier and more active than usual. However, the sample was reasonably representative of national weight status and socio-economic characteristics. Even with this heavily adjusted longitudinal design, establishing causality is not possible and findings should be regarded as providing supportive rather than definitive evidence.

## Conclusions

Moderate-to-vigorous physical activity is associated with subsequent unplanned hospital admissions and numbers of prescriptions in adults in their 70 s and 80 s, over a four to five year period. These results are independent of many potentially confounding socio-economic factors, weight status, the presence of existing disease, and in the case of numbers of prescriptions, objectively assessed lower limb function. The data provides support for greater availability of community-based programs to increase physical activity and prevent loss of lower limb function, and suggests the potential for health care cost savings.
